# Psychometric evaluation of a Korean-language version of the Nursing Home Survey on Patient Safety Culture (K-NHSPSC)

**DOI:** 10.1186/s12912-025-04243-x

**Published:** 2025-12-19

**Authors:** Seung Eun Lee, So Young Park

**Affiliations:** 1 College of Nursing, Mo-Im KIM Nursing Research Institute, Yonsei University, 50-1 Yonsei-ro, Seodaemun-gu, Seoul, 03722 South Korea; 2 Department of Psychology, Yonsei University, 50-1 Yonsei-ro, Seodaemun-gu, Seoul, 03722 South Korea

**Keywords:** Korea, Nursing homes, Organizational culture, Psychometrics, Reliability, Safety management, Validity

## Abstract

**Background:**

Patient safety culture is a foundational component of quality care in long-term care facilities. Although the Nursing Home Survey on Patient Safety Culture (NHSPSC) has been widely used and validated internationally, there has been no validated Korean version applicable to the Korean context. This study aimed to translate, culturally adapt, and validate the NHSPSC for use in Korean long-term care settings.

**Methods:**

We employed a two-phase design. In Phase 1, the original NHSPSC was translated into Korean using a committee-based translation method, followed by an expert review for content validity. A panel of five experts rated the relevance and clarity of each item. Subsequently, cognitive interviews were conducted with five nursing home staff to assess item comprehension and cultural appropriateness. In Phase 2, a cross-sectional survey was administered to healthcare workers in 10 nursing homes. After applying exclusion criteria, data from 282 participants were analyzed. Internal consistency was assessed using Cronbach’s alpha. Construct validity was evaluated through confirmatory factor analysis and correlations with global safety ratings.

**Results:**

The Korean version of the NHSPSC retained all 12 original factors and demonstrated acceptable internal consistency. Confirmatory factor analysis supported the factorial structure of the instrument. Correlations between subscales and global safety ratings provided additional support for construct validity.

**Conclusions:**

The Korean version of NHSPSC demonstrated acceptable psychometric properties and retains the original instrument’s multidimensional structure. It is a reliable and valid tool for assessing patient safety culture in Korean nursing homes and can facilitate both domestic quality improvement and international benchmarking efforts.

## Background

As of late 2025, South Korea has become a super-aged society, with individuals aged 65 and older accounting for more than one-fifth of the total population. This proportion is expected to rise further, reaching approximately 44% by 2050 [1]. The nation’s response to this unprecedented demographic shift will play a critical role in shaping the future of public health and long-term care infrastructure [1]. In light of the rapidly aging population, the Korean government introduced the Long-Term Care Insurance (LTCI) system in 2008 to alleviate the caregiving burden on families and support older adults with increasing care needs. Since its inception, the program has significantly expanded both scope and scale. As of December 2023, Korea’s LTCI system served over 1 million beneficiaries through more than 6,200 institutional care providers [2].

However, as the number of institutionalized residents increased, concerns have grown regarding safety and quality of care. Recent systematic reviews of international studies have identified adverse events—such as falls and medication errors—as common and consequential risks in long-term care settings [3]. Although most of this evidence comes from outside Korea, it underscores the importance of examining resident safety in the Korean context as well. Establishing a robust safety culture is widely recognized as a foundational strategy for reducing adverse events and promoting high-quality care [4]. In healthcare, safety culture is understood as the shared values, beliefs, and norms within a work setting that shape staff behaviors and attitudes related to safety [5]. While initiatives to strengthen safety culture are increasingly common in hospital settings [6], long-term care facilities face unique challenges stemming from differences in staffing, organizational structure, and models of care delivery [7]. These differences highlight the need for measurement instruments that are appropriately tailored to characteristics of long-term care.

In 2008, the Agency for Healthcare Research and Quality (AHRQ) developed the Nursing Home Survey on Patient Safety Culture (NHSPSC) to assess safety culture specifically within nursing home environments [8]. Since its release, the NHSPSC has been translated, culturally adapted, and psychometrically validated in several countries, including China [9], Indonesia [10], Spain [11], and Switzerland [12]. While its international application continues to expand, no cross-culturally validated version of the NHSPSC currently is available in the Korean long-term care sector. Instead, Yoon and colleagues [13] developed a context-specific instrument through literature review and expert panel evaluation. Although meaningful within the Korean setting, this tool was newly constructed rather than adapted from internationally established instruments such as the NHSPSC, and its validation relied on exploratory factor analysis without cross-cultural testing, limiting its potential for international benchmarking or policy comparison. This gap is further underscored by a recent systematic review of 25 studies on safety culture measurement in nursing homes, which found no Korean-language application of the NHSPSC [7].

Beyond this measurement gap, safety culture needs to be interpreted within its broader sociocultural context. A systematic review on patient safety culture in healthcare noted that East Asian countries share cultural norms, values, and beliefs that differ from those in Western settings, which may influence employees’ perceptions of safety culture [14]. These findings suggest that Western-developed instruments such as the NHSPSC require careful cultural adaptation to ensure conceptual and contextual equivalence in East Asian settings.

To address this gap, the present study aimed to adapt the NHSPSC for use in Korean nursing homes through psychometric validation. Developing a culturally appropriate yet internationally comparable instrument is essential for accurately assessing patient safety culture in Korea’s long-term care sector. Recent international initiatives—such as the Organization for Economic Co-operation and Development (OECD)’s cross-national assessment of safety culture in hospital settings [15]—highlight the increasing importance of validated tools for benchmarking safety performance across countries. Such instruments can help improve care quality within national systems and enable meaningful international comparisons that inform evidence-based policy development.

## Methods

### Study design

This study employed a two-phase approach to adapt and validate the AHRQ’s NHSPSC in the Korean context. In the first phase, the original instrument was translated into Korean using a committee-based method [5]. A panel of bilingual professionals reviewed the translation to assess item clarity, semantic equivalence, and cultural appropriateness. In the second phase, the psychometric properties of the Korean version were evaluated using data collected from nursing home staff. This included analyses of internal consistency and confirmatory factor analysis to examine construct validity.

### Instrument

The NHSPSC is based on the conceptualization of patient safety culture in long-term care as the shared values, beliefs, and behavioral norms that shape staff actions and organizational practices related to resident safety. The original instrument comprises 42 items grouped into 12 dimensions of safety culture: teamwork, staffing, compliance with procedures, training and skills, nonpunitive response to mistakes, handoffs, feedback and communication about incidents, communication openness, supervisor expectations and actions promoting resident safety, overall perceptions of resident safety, management support for resident safety, and organizational learning [8]. Each item is rated on a 5-point Likert scale, either by level of agreement (1 = *strongly disagree* to 5 = *strongly agree*) or frequency (1 = *never* to 5 = *always*), depending on the item. In addition, the survey includes two single-item measures of overall safety in the nursing home. One item evaluates whether respondents perceive the facility as safe enough to recommend to friends or family, using a 3-point response scale (1 = *Yes*, 2 = *Maybe*, 3 = *No*). The other item asks for a global assessment of resident safety, rated on a 5-point scale ranging from 1 (*Poor*) to 5 (*Excellent*).

### Translation process

After obtaining permission from the AHRQ, we initiated the translation of the NHSPSC into Korean. A committee-based translation approach was employed to ensure cultural and linguistic equivalence, particularly given the substantial differences between English and Korean [5]. The translation committee consisted of four bilingual Korean nurse professionals: two with clinical experience in both the United States and South Korea, one with prior experience working in a Korean nursing home, and another with substantial research experience focused on Korean nursing home environments. Initially, each committee member independently translated the survey items. The committee then convened to review the translations, resolve discrepancies, and refine ambiguous expressions. For example, the English term “resident” required careful deliberation because commonly used Korean equivalents differ in tone—some carry a more honorific nuance, whereas others are more administrative or institutional. The committee selected wording that would be culturally appropriate while remaining consistent with terminology used in Korean nursing homes. Through discussion and consensus, a preliminary Korean version was established.

### Expert review

After completing the translation, we engaged a panel of five experts to evaluate the cultural relevance and clarity of each item. The panel consisted of two researchers conducting research on nursing homes, one nursing home director, one nursing home staff, and two individuals responsible for evaluating long-term care facilities. Each panelist independently rated the appropriateness and clarity of the translated items on a four-point relevance scale (1 = *Not relevant* to 4 = *Highly relevant*). We computed item-level content validity indices (I-CVI) by calculating the proportion of experts who rated each item as either 3 or 4 and calculated the average of these scores to obtain the scale-level CVI (S-CVI). I-CVI scores exceeding 0.80 were regarded as acceptable, while an S-CVI above 0.90 indicated excellent content validity. Based on this process, minor refinements were made to a few items, and the wording of selected phrases was adjusted to improve cultural and contextual clarity [16].

### Cognitive interview

Following the expert review, we conducted semi-structured, face-to-face cognitive interviews with five nursing home staff members from diverse roles (i.e., a social worker, a nursing assistant, a care helper, a staff nurse, and a nurse manager). These interviews aimed to assess participants’ comprehension of the items, response options, and survey instructions. Participants were also asked to suggest revisions for any unclear wording or confusing terminology. Based on their feedback, minor revisions were made, and the final version of the K-NHSPSC was established through this iterative refinement process.

### Setting, sample, and data collection

Using a convenience sampling approach, we recruited participants from 10 nursing homes located in three cities in South Korea. Data collection took place between August and December 2024, using paper-based questionnaires administered on-site. Participants were eligible if they were currently employed at a nursing home and had at least three months of work experience. A total of 351 questionnaires were distributed, and 333 participants completed the survey (response rate = 94.9%). Across the 10 participating nursing homes, the number of respondents per facility ranged from 6 to 52, depending on staffing levels and staff availability during data collection. After excluding six participants with less than three months of experience; 34 who either did not report their employment duration or failed to respond to key patient safety culture items; and 11 with highly uniform response patterns (e.g., over 80% identical answers), which we treated as a form of insufficient effort responding in line with prior research on low-effort responding [17], the final analytic sample consisted of 282 participants.

Although determining the ideal sample size for Confirmatory Factor Analysis (CFA) is influenced by factors such as the number of latent variables, the number of indicators per factor, and the expected factor loadings, previous guidelines recommend a sample-to-item ratio between 5:1 and 20:1 [18], with minimum sample sizes ranging from 100 to 200 [19]. Based on a conservative 5:1 ratio for the 42-item instrument, we estimated that at least 210 participants would be required and therefore aimed to recruit more than 250 participants to allow for missing data and exclusions. Given the 42-item structure of the instrument, the final sample size of 282 (approximately 6.7 participants per item) was considered sufficient for conducting CFA and evaluating the instrument’s factor structure.

### Data analysis

Descriptive analyses were first conducted to examine the demographic characteristics of the sample and the distribution of responses across survey items. Negatively worded items were reverse-coded so that higher scores consistently reflected more positive perceptions of patient safety. Internal consistency of each subscale was evaluated using Cronbach’s alpha coefficients. These analyses were performed using IBM SPSS Statistics version 30. Structural validity was assessed through CFA, using Mplus version 8.11 [20]. As the NHSPSC is a theory-driven instrument with an established 12-factor structure, we conducted CFA with confirmed 12 factors [21, 22]. Model fit was evaluated using several indices, including chi-square (χ²), comparative fit index (CFI), Tucker–Lewis index (TLI), and root mean square error of approximation (RMSEA). Acceptable model fit was as CFI and TLI ≥ 0.80 or higher [23] and RMSEA ≤ 0.06 [24]. To examine convergent validity, correlations were calculated among the 12 subscales as well as between the 12 subscales and the two global safety items. Pearson’s correlation coefficients were applied for correlations among the subscales, whereas Spearman’s rho was used for correlations with the global safety items, since these were considered ordinal data [25]. A *p*-value of < 0.05 was considered statistically significant.

## Results

### Participants’ characteristics

As shown in Table 1, nearly half of the participants were care helpers (53.9%), followed by nurses (16.7%), social workers (8.5%), and nursing assistants (5.3%). Most participants (89.6%) were female, with a mean age of 53.2 years (*SD* = 10.3). The average length of tenure in the current nursing home was 5.9 years (*SD* = 6.0). Regarding educational background, most participants had either a high school diploma (39.9%) or a bachelor’s degree (36.0%). Most participants were directly involved in resident care (83.6%), employed full-time (92.5%), and held permanent positions (70.2%).

**Table 1:** Participant characteristics (*N* = 282)

Characteristic	*n*	%	Mean	SD
Job category				
Facility Administrator	2	0.7		
Assistant Administrator	7	2.5		
Nurse	47	16.7		
Social worker	24	8.5		
Nurse assistant	15	5.3		
Care helper	152	53.9		
Therapist	10	3.5		
Nutritionist	2	0.7		
Administration staff	11	3.9		
Other assistant staff	12	4.3		
Gender				
Male	26	10.4		
Female	225	89.6		
Age (years)			53.2	10.3
Education				
Highschool diploma	111	39.9		
Associate degree	40	14.4		
Bachelor’s degree	100	36.0		
Master’s degree or higher	27	9.7		
Tenure in current nursing home (years)			5.9	6.0
Employment contract type				
Permanent	198	70.2		
Temporary	84	29.8		
Employment status				
Full-time	259	92.5		
Part-time	21	7.5		
Shift				
Fixed shift	117	43.0		
Two-shift rotation	49	18.0		
Night	106	39.0		
Direct care involvement				
Yes	230	83.6		
No	45	16.4		

Note. *SD* = Standard Deviation

Percentages are based on valid responses. Missing data: gender (*n* = 31), education (*n* = 4), employment status (*n* = 2), shift (*n* = 10), direct care involvement (*n* = 7)

### Reliability

The internal consistency reliability of the K-NHSPSC was assessed using Cronbach’s alpha coefficients for each of the twelve subscales. As presented in Table 2, Cronbach’s alpha values ranged from 0.58 to 0.87. While the Nonpunitive Response to Mistakes subscale yielded a relatively low alpha value (α = 0.58), this falls within the range considered acceptable for exploratory research [26].

**Table 2:** Factor loadings, means, standard deviations, and cronbach’s alphas of the 12 factors

Factor	Item number from original NHSPSC	Factor Loading	*Mean*	SD	Cronbach’s alpha
F1	A1	0.77	3.82	0.65	0.86
	A2	0.73			
	A5	0.85			
	A9	0.62			
F2	A3	0.72	3.29	0.65	0.60
	A8	0.44			
	A16	0.34			
	A17	0.67			
F3	A4	0.80	3.94	0.61	0.65
	A6	0.39			
	A14	0.50			
F4	A7	0.60	4.01	0.63	0.71
	A11	0.73			
	A13	0.70			
F5	A10	0.49	3.23	0.65	0.58
	A12	0.50			
	A15	0.51			
	A18	0.53			
F6	B1	0.66	4.11	0.61	0.85
	B2	0.78			
	B3	0.72			
	B10	0.82			
F7	B4	0.72	4.13	0.58	0.86
	B5	0.85			
	B6	0.74			
	B8	0.77			
F8	B7	0.86	3.70	0.73	0.78
	B9	0.70			
	B11	0.68			
F9	C1	0.91	4.02	0.71	0.87
	C2	0.79			
	C3	0.71			
F10	D1	0.76	4.28	0.58	0.84
	D6	0.81			
	D8	0.80			
F11	D2	0.73	4.10	0.66	0.84
	D7	0.85			
	D9	0.79			
F12	D3	0.41	3.82	0.59	0.73
	D4	0.59			
	D5	0.76			
	D10	0.79			

Note. *Mean*,* SD*, and Cronbach’s α values refer to factor-level statistics, not item-level

NHSPSC = Nursing Home Survey on Patient Safety Culture; SD = Standard Deviation; F1 = Teamwork; F2 = Staffing; F3 = Compliance with Procedures; F4 = Training and Skills; F5 = Nonpunitive Response to Mistakes; F6 = Handoffs; F7 = Feedback and Communication About Incidents; F8 = Communication Openness; F9 = Supervisor Expectations and Actions Promoting Resident Safety; F10 = Overall Perceptions of Resident Safety; F11 = Management Support for Resident Safety; F12 = Organizational Learning

### Content validity (phase 1 results)

All translated items were reviewed by a panel of five experts to evaluate their cultural relevance and linguistic clarity. The I-CVI scores ranged from 0.80 to 1.00, suggesting that most items were considered as highly relevant. During the expert review, only one item—“Staff use shortcuts to get their work done faster”—received an I-CVI of 0.80. Experts noted that the literal translation of “shortcuts” was too abstract and could be interpreted in multiple ways within the Korean long-term care context, making the intended meaning unclear. The original item refers specifically to staff bypassing or not fully adhering to standard procedures to complete tasks more quickly. Therefore, the wording was revised to explicitly reflect “not following standard procedures,” which provides clearer conceptual meaning and improves cultural appropriateness. The S-CVI was 0.99, indicating excellent overall content validity. These results support the appropriateness of the translated items for use in the Korean nursing home context.

### Construct validity (phase 2)

All standardized factor loadings were statistically significant and ranged from 0.34 to 0.91, exceeding the commonly accepted minimum threshold of 0.30 [27], thereby supporting the proposed measurement structure (see Table 2). As shown in Table 3, the one-factor model demonstrated poor fit to the data [χ²(819) = 2688.95, *p* < .001, CFI = 0.71, TLI = 0.70, RMSEA = 0.09], indicating that a unidimensional structure did not adequately represent the data. In contrast, the hypothesized twelve-factor model showed a substantially improved fit [χ²(753) = 1542.18, CFI = 0.88, TLI = 0.86, RMSEA = 0.06]. These findings provide empirical support for the multidimensional structure of the instrument, thereby supporting its construct validity [21].

**Table 3:** Fit indices for confirmatory factor analyses

	χ2	df	χ2/df	CFI	TLI	RMSEA
One-factor model	2688.95*	819	3.28	0.71	0.70	0.09
Twelve-factor model	1542.18*	753	2.05	0.88	0.86	0.06

Note. df = degrees of freedom; CFI = Comparative Fit Index; TLI = Tucker-Lewis Index; RMSEA = Root Mean Square Error of Approximation. * *p* < .001

Intercorrelations among the 12 subscales ranged from 0.26 to 0.77 (*p* < .01; see Table 4). Although some subscale pairs showed strong correlations (e.g., Management Support for Resident Safety and Organizational Learning: *r* = .74), all remained below the commonly cited threshold of 0.80, supporting adequate discriminant validity and conceptual distinction among the constructs [25]. To further assess construct validity, Spearman’s rho correlations were calculated between the 12 subscales and two overall safety ratings [25]. All subscales were significantly and positively correlated with both global safety items: one reflecting the respondent’s willingness to recommend the facility to others, and the other assessing the overall resident safety (*ρ* = 0.23–0.67, *p* < .01). These findings provide evidence of convergent validity, suggesting that higher safety culture subscale scores are associated with more global assessments of safety.

**Table 4:** Correlations among the 12 subscales and two global safety items

	F1	F2	F3	F4	F5	F6	F7	F8	F9	F10	F11	F12	E1
F1													
F2	0.45												
F3	0.51	0.42											
F4	0.57	0.51	0.58										
F5	0.42	0.43	0.37	0.43									
F6	0.53	0.41	0.49	0.61	0.38								
F7	0.59	0.50	0.47	0.60	0.40	0.74							
F8	0.56	0.51	0.44	0.57	0.46	0.55	0.67						
F9	0.63	0.40	0.49	0.58	0.39	0.57	0.69	0.65					
F10	0.51	0.44	0.50	0.54	0.26	0.60	0.67	0.58	0.57				
F11	0.58	0.44	0.47	0.61	0.32	0.63	0.71	0.69	0.69	0.77			
F12	0.43	0.45	0.49	0.55	0.34	0.60	0.65	0.60	0.57	0.72	0.74		
E1	0.34	0.29	0.23	0.32	0.25	0.37	0.40	0.40	0.31	0.51	0.47	0.48	
E2	0.46	0.34	0.33	0.49	0.23	0.44	0.50	0.48	0.43	0.67	0.57	0.55	0.54

Note. F1 = Teamwork; F2 = Staffing; F3 = Compliance with Procedures; F4 = Training and Skills; F5 = Nonpunitive Response to Mistakes; F6 = Handoffs; F7 = Feedback and Communication About Incidents; F8 = Communication Openness; F9 = Supervisor Expectations and Actions Promoting Resident Safety; F10 = Overall Perceptions of Resident Safety; F11 = Management Support for Resident Safety; F12 = Organizational Learning; E1 = Recommend Facility; E2 = Overall Safety Rating. All factor loadings were statistically significant at *p* < .01

## Discussion

This study aimed to address the lack of validated instruments for assessing patient safety culture in Korean nursing homes by translating and culturally adapting the AHRQ’s NHSPSC. Overall, through rigorous psychometric testing, the Korean version of the NHSPSC demonstrated acceptable internal consistency and a stable multidimensional factor structure.

Our CFA of the K-NHSPSC supported the original 12-factor structure, demonstrating a stable multidimensional design of the measurement in the Korean context. While previous cross-cultural validations—such as the Chinese [9], French [28], Indonesian [10], Norwegian [29], Spanish [11], and Swiss [12] versions—also maintained a multidimensional structure, they reported reduced factor structures, typically retaining between 4 and 10 subscales. In contrast, our results align with the Polish adaptation by Switalski et al. [25], which also retained all 12 original dimensions. This consistency supports the conceptual robustness of the original instrument and provides evidence for the construct validity and cross-cultural applicability of the K-NHSPSC.

In addition to supporting structural validity, we also examined the internal consistency of each subscale to assess the reliability of the K-NHSPSC. Based on commonly accepted criteria for translated or adapted instruments (α ≥ 0.60) [30, 31], all subscales met the threshold, with Cronbach’s alpha coefficients ranging from 0.60 to 0.87—except for the Nonpunitive Response to Mistakes subscale, which yielded an alpha of 0.58. Although slightly below the threshold, this subscale was retained due to its theoretical importance. Previous studies have also suggested that alpha values above 0.58 may be acceptable in the early stages of instrument adaptation or for subscales with fewer items [26]. Notably, the Spanish version of the NHSPSC also reported a low alpha (0.479) for this same subscale [11]. Lower internal consistency may reflect the small number of items included or the conceptual heterogeneity inherent in the construct [32]. Future research should continue to examine the reliability of this subscale in diverse nursing home settings and with larger samples to determine whether the internal consistency can be improved or remain stable across contexts.

Although several subscales in the present study—such as Overall Perceptions of Resident Safety, Management Support for Resident Safety, and Organizational Learning—showed moderately high intercorrelations (*r* = 0.72–0.77), all values remained below the accepted threshold of 0.80, indicating acceptable discriminant validity and conceptual distinction among the constructs [25]. Nonetheless, similar patterns have been observed in prior cross-cultural validations of the NHSPSC. For example, in the Norwegian version [29], the three subscales showed high intercorrelations (*r* ≥ .90), which led to the merging of these dimensions into a single factor—a tendency also reported in the Spanish [11] and Swiss [12] versions. This convergence suggests that in nursing home settings, staff may perceive these elements as part of a broader, unified safety climate, especially where managerial engagement and continuous learning are closely tied to perceptions of resident safety. Given the relatively high intercorrelations observed in our study and in prior cross-cultural validations, future research could further investigate the dimensional structure of these subscales in nursing home settings.

By adapting the NHSPSC to the Korean context and validating its psychometric properties, this study provides a reliable instrument for both domestic quality assessment and global benchmarking. Findings indicate that the K-NHSPSC retains the original 12-factor structure and demonstrates acceptable levels of internal consistency and construct validity. In addition, the expert review and cognitive interviewing processes support the cultural appropriateness of the K-NHSPSC. Experts rated nearly all items as highly relevant to the Korean nursing home context, and staff participating in cognitive interviews reported that the translated items were understandable and reflected safety-related practices, communication patterns, and organizational dynamics common in Korean facilities. These quantitative and qualitative findings suggest that the K-NHSPSC is both statistically robust and culturally appropriate for use in Korean long-term care settings. These properties make the instrument a reliable tool for assessing safety culture in Korean nursing homes and ensure conceptual alignment with the original AHRQ version, thereby allowing for meaningful cross-national comparisons. In light of recent OECD benchmarking initiatives in patient safety culture [15], the availability of a rigorously validated, culturally adapted survey instrument represents a significant contribution to both research and policy.

### Limitations

This study has several limitations that should be noted. The sample was drawn from a limited number of facilities and may not fully represent all Korean nursing homes. Future research should validate the instrument’s psychometric properties in more diverse and representative nursing home settings to ensure its broader applicability. In addition, the cross-sectional design precludes causal interpretations. Future studies should examine the survey’s predictive validity and test-retest reliability and consider how safety culture perceptions relate to actual patient safety outcomes in nursing home settings. Finally, although the instrument was rigorously translated and reviewed, subtle cultural or linguistic nuances may influence how items are interpreted.

## Conclusions

This study translated, culturally adapted, and validated the NHSPSC for use in Korean long-term care settings. The K-NHSPSC retained the original 12-factor structure and demonstrated acceptable levels of internal consistency and construct validity. Overall, the study findings suggest that the instrument offers a sound and valid approach for evaluating how staff perceive safety culture within nursing home settings. Given its structural alignment with the original AHRQ tool, the K-NHSPSC can serve as a foundation for national quality improvement efforts and international benchmarking. Future research should continue to explore its applicability across diverse care settings and examine its predictive validity in relation to safety outcomes.

## Data Availability

The datasets generated and/or analyzed during the current study are not publicly available due to them containing information that could compromise research participant consent but are available from the corresponding author on reasonable request.

## Abbreviations

AHRQ: Agency for Healthcare Research and Quality
NHSPSC: Nursing Home Survey on Patient Safety Culture
K-NHSPSC: Korean version of Nursing Home Survey on Patient Safety Culture
